# Assessment of C Fibers Evoked Potentials in Healthy Subjects by Nd : YAP Laser

**DOI:** 10.1155/2022/7737251

**Published:** 2022-12-26

**Authors:** Elena Ammendola, Giusy Tancredi, Katia Ricci, Giovanni Falcicchio, Massimiliano Valeriani, Marina de Tommaso

**Affiliations:** ^1^Neurophysiolopathology Unit, Bari Aldo Moro University, Bari, Italy; ^2^Department of Neuroscience, Headache Center, Bambino Gesù Children's Hospital, Istituto di Ricovero e Cura a Carattere Scientifico (IRCCS), Rome, Italy; ^3^Center for Sensory-Motor Interaction, Aalborg University, Aalborg, Denmark

## Abstract

**Introduction:**

Although laser stimuli activate both Ad- and C-fibres, the corresponding laser evoked potentials (LEPs) remain restricted to the Ad-fibers input, while the C-fibers related potential is hardly detectable.

**Aims:**

To evaluate multichannel ultralate LEPs (U-LEPs) by using Nd : Yap laser pulses in healthy volunteers to stimulation of face and lower and upper limbs, in order to estimate the reliability of C-LEPs elicited from both trigeminal and somatic sites.

**Methods:**

Twenty healthy volunteers participated in two stimulation sessions to record A*δ*-LEPs and C-LEPs. We used a Nd : YAP Laser and 62 EEG recording electrodes. Stimuli parameters were set to activate either small myelinated (A*δ*), eliciting purely warmth sensations, or unmyelinated (C) afferents, and eliciting pinprick sensations.

**Results:**

At the trigeminal level, we obtained a negative-positive complex in a time interval compatible with the C fibers activation. In the somatic districts, the averaged responses consisted of an earlier negative-positive complex, followed by a later one. Single trials analysis of U-LEPs showed a maximal positive peak in a time interval in the range of C fibers. Topographical analysis of U-LEPs resembled that of LEPs. All subjects exhibited readable U-LEPs in at least 2 stimulated sites. *Discussion*. A purely warmth sensation seems to correspond to A*δ* and C-fibers coactivation, at least in the somatic districts. While the related cortical waves seem hardly readable, their total absence could be a sign of systemic involvement of warm related C-fibers in specific clinical conditions.

## 1. Introduction

Laser evoked potentials (LEPs) are a reliable method for nociceptive pathways study. Application in clinical practice is based on a-delta fibers stimulation, which evokes clear potentials, generated within main cortical areas devoted to pain processing, such as SII, insula, and anterior cingulate [1].

Laser stimulators determine concurrent activation of a-delta and C nociceptors. The late cortical responses induced by C fibers are masked by the previous activation of the faster fibers, according to the physiological properties of the cortex as “first comes, first serves” [2]. However, previous studies demonstrated that selective activation of C fibers could be obtained with specific LEP recording modalities [3] elicited ultralate LEPs (U-LEPs) from the hand, using a thulium laser on a tiny surface skin area (0.23 mm). Such a method is based on the higher density of C nociceptors as compared to a-delta nociceptors in the skin [4]. Despite the U-LEPs were obtained in allthe 9 healthy subjects, an earlier complex appeared as a correlate of A*δ* fibers recruitment. In that study, the motor reaction to laser stimulation was compatible with nonmyelinated fiber stimulation. Many subjects experienced a tactile sensation, in accordance with the activation of polymodal or mechano heat responsive C-fibers [5, 6]. Kakigi et al. confirmed that most of the subjects stimulated with a tiny laser stimulus diameter, felt in general touch or pressure sensation or slight burning pain, probably due to the activation of C nociceptors [7].

Another method to evaluate the activation of C fibers used the neodymium : yttrium-aluminium-perovskite laser (Nd : YAP). YAP laser pulses of low intensity (16 ± 37 mJ/mm^2^), relatively-long duration (10 ms), and large irradiated area (∼180 mm^2^), raising the temperature of the facial skin to 39°C [8]. This modality of stimulation elicits a pure warmth sensation, compatible with warm responsive C-fibers. Furthermore, studies reported C-related clear responses in the trigeminal areas, which originated in similar cortical sources as the A*δ* related potentials [9, 10], while responses from the somatic site have been rarely examined, for the dispersion of the small potentials obtained from the distal sites of stimulation, and their consequent scarce reliability.

The aim of the present study is to record multichannel U-LEPs by using Yap laser pulses in healthy volunteers to stimulation of not only facial districts but also lower and upper limbs, in order to estimate their reliability from both trigeminal and somatic sites.

## 2. Methods

### 2.1. Subjects

Twenty healthy volunteers (7 male and 13 female), aged 22–64 years, participated in two stimulation sessions to record A*δ*-LEPs and C-LEPs. We choose a wide age range, excluding younger cases, in accordance with the substantial stability we observed for CO^2^ trigeminal LEPs in subjects older than 18 years, and the small differences of somatic LEPs between the 2^th^-3^th^ and the 4^th^–6^th^ decades [11]. They were selected based on no objective sign and/or history for general medical, neurological, and psychiatric disorders, including vitamins deficit, thyroid disease, active viral infections, and absence of in-course or previous use of CNS acting drugs. All subjects gave informed consent, and the local Ethical Committee of the Policlinico General Hospital approved the study.

### 2.2. Stimulation

We used a neodymium : yttrium-aluminium-perovskite laser (Nd : YAP) stimulator with fibre-optic guidance, produced by Electronic Engineering ®, Florence, Italy. Laser stimuli parameters were set to activate either small myelinated (A*δ*) or unmyelinated (C) afferents [9] (A*δ* modality or C modality). Laser stimuli were applied on the right side of the body, at the level of the first branch of the trigeminal nerve, dorsum of the hand, knee (middle and lateral sides), and dorsum of the foot. The site of stimulation was moved slightly for each stimulus to avoid habituation and skin damage. All subjects received at least 25 consecutive stimulations, for each site and for both type of potentials (A*δ*- and C-LEPs) with an interstimulus interval (ISI) of at least 10–15 seconds. The total duration of the procedure, taking into account the subject information, the consent signing, the EEG montage, the stimulation setting assessment, the time of a single stimulation session, and the intersession intervals, was around 100 min, so we preferred to limit the evaluation to the right side, also in accord with previous studies of our group in healthy subjects [11]. Subjects were instructed to take attention to single stimuli, in order to estimate the subjective sensation of pain for the A*δ* modality of stimulation and the sensation of innocuous warmth for the C modality of stimulation. During the C-modality of stimulation, all subjects were advised to alert the technician after each stimulus evoking a sensation different from such warmth, as a pinprick sensation or burning pain.

Nd : YAP laser pulses of low intensity (2.23–13.37 J/cm^2^), relatively long duration (10 ms), and large irradiated area (diameter 10 mm) were used to elicit purely warmth sensations, while pulses of higher intensity (8.91–25.46 J/cm^2^), shorter duration (5 ms), and small irradiated area (diameter 5 mm) were used to elicit pinprick sensations. The individual detection threshold was determined by up-regulating the energy stepwise (one step is 0.25 J) until the subjects felt any sensation. For the A*δ* modality of stimulation, from the detection threshold, the energy was up-regulated further until the subjects reported a distinct pinprick pain sensation between 3 and 6 on the numerical pain rating scale (0 = no pain, 10 = most imaginable pain) which was equal to a twofold detection threshold [12].

Regarding A*δ*-LEPs, the laser energy density which induced a painful pinprick sensation ranged from 8.91 to 17.83 J/cm^2^ (1.75–3.50 J) in the territory of the first branch of the trigeminal nerve, from 10.19 to 20.37 mJ/mm^2^ (2.00–4.00 J) at the dorsum of the hand, from 11.46 to 20.37 J/cm^2^ (2.25–4.00 J) at the knee, and from 11.46 to 25.46 J/cm^2^ (2.25–5.00 J) at the dorsum of the foot. Following each trial, a visual-analogue scale (VAS) was presented. Lower (VAS = 0) and upper (VAS = 100) extremities of the scale were labeled “No perception” and “Maximum pain.” Regarding C-LEPs, the laser energy density which induced purely warmth sensations ranged from 2.55 to 10.50 J/cm^2^ (2.00–8.25 J) in the territory of the first branch of the trigeminal nerve, from 2.23 to 11.14 J/cm^2^ (1.75–8.75 J) at the dorsum of the hand, from 2.55 to 12.41 J/cm^2^ (2.00–9.75 J) at the knee, and from 6.37 to 13.37 J/cm^2^ (5.00–10.50 J) the at dorsum of the foot (Tables 1 and 2). The diameter of the illuminated area was measured with near-infrared-sensitive paper and was kept at 5 mm for A*δ*-LEPs and at 10 mm for C-LEPs.

### 2.3. Recording

Subjects were seated in a comfortable chair and wore protective goggles. They were instructed to keep their eyes open and gaze slightly downwards. EEG was recorded from 64 Ag–AgCl electrodes placed on the scalp by a prewired head cap, according to the International 10–20 system. An electrode placed on nasion was used as a reference. Ground was placed at the right forearm. Two electrodes placed at the upper left and lower right side of the right eye monitored ocular movements and eye blinks. Impedance was kept below 5 kΩ. LEPs were recorded using a MICROMED EEG apparatus (Micromed Brain Quick, Mogliano Veneto, Italy), through a 1.6–70 Hz bandwidth, with an analysis time of 2000 ms.

### 2.4. LEPs Analysis

We conducted preprocessing in MATLAB using the EEGLAB 14_1_1 tool, according to previous studies [13]. The data were initially high-pass filtered at 1 Hz to remove the slow drifts. Subsequently, we applied a notch filter at 50 Hz (L: 48, H: 52) to remove the power line noise artefacts. Activities exceeding 150 *μ*V of amplitude were automatically removed. To precompute the channel measures, we deleted the EOG-related artefact components of the independent component analysis and performed a spherical interpolation of the missing channels. Bad channels were identified using a semiautomatic method, based on visual detection and channel statistics. Channels presenting the distributions of potential values farther from the Gaussian distribution were removed. We precomputed the LEPs in the time interval of 2000 ms poststimuli, using a 70 Hz low-pass filter, removing the baseline and considering the 100 ms preceding the laser stimulus. For all subjects, we averaged 21 artefact-free trials for each stimulation site. We used the LetsWave tool version 7. Following the visual analysis of individual averaged track, we identified major A*δ*-LEP waves. In the present study, taking into consideration the low amplitude of C-related potentials, we focused the analysis on the vertex N2-P2 components. For the A*δ*-related potentials, we identified the N2-P2 complex in the interval 180–450 msec. For the C-related potentials, we visually identified the main peaks. In the cases in which there was on average a negative-positive response at the vertex, clearly identifiable from the signal noise, we established the maximum negativity and positivity in the interval 200–2000, just to include those potentials eventually evoked by A*δ*-fibers coactivation. For this analysis, the Cz channel referred to as the nasion was considered. Thus, the amplitudes and latencies of LEPs were measured at the maximal peak in the predetermined interval, considering the average and the single trials.

Conduction velocity was measured by using the P2 peak latency, considering the distance between the foot and the knee for the lower limb. At the trigeminal level, we took into consideration previous reports with the analogous modality of stimulation [9]. Topographical analysis of main peaks was obtained using the LetsWave vers. 7 software.

### 2.5. Statistical Method

Latency, amplitude (measured on Cz derivation), and laser intensity related to the average of A*δ* and C fibers evoked potentials, were compared using the Student's *t* test for paired data. With alpha 0.05, and 30% differences between a-delta and C related responses latencies and amplitudes, we had a power of 0.97%. In order to evaluate the possible coactivation of A*δ* fibers during the C fibers modality of stimulation, we considered the latencies of the maximal positive peak of the single responses and compared them after both stimulation modalities using the repeated measures ANOVA with the stimulations modality as a factor.

## 3. Results

All subjects reported a clear painful pinprick sensation for A*δ*-stimulation modality, and a burning not painful sensation for C-fibers stimulation. In Table 1, the stimulation parameters are reported for individual subjects for the study of A*δ*-fibers, while Table 2 shows the stimulation parameters for the study of C-fibers. One patient did not show ultralate LEPs (ULeps) in two sites (foot and hand), the others had absent ULeps only to the stimulation of one site (Table 2). Females reported lower pinprick threshold in all the stimulated sites as compared to males (Student's *t* test <0.05), while the warmth threshold was similar between the two sexes.

### 3.1. Trigeminal District

**Figure 1:**
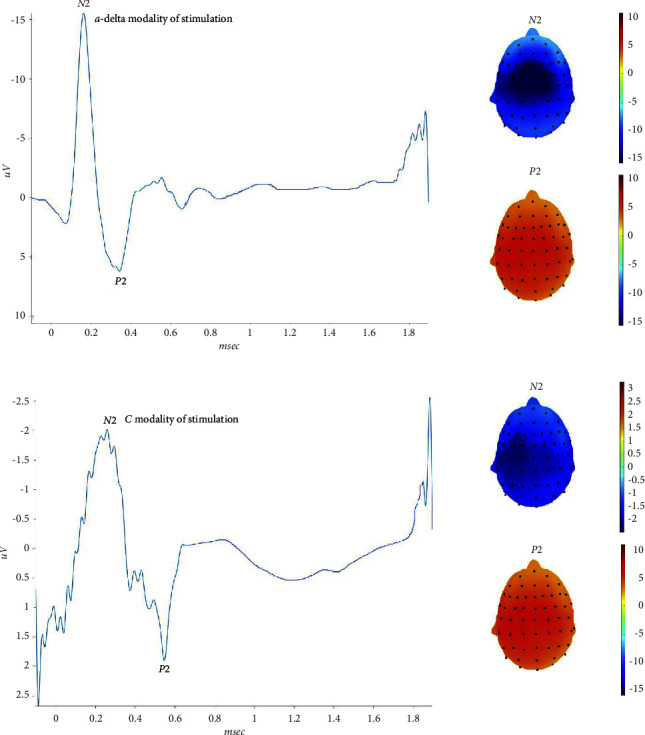
On the left side, the grand average of N2 P2 waves obtained by the right first trigeminal branch stimulation are reported. On the right side, the corresponding topographical maps are represented. Upper: a-delta modality of stimulation. Lower: C-modality of stimulation.

In the trigeminal district, all subjects presented with clear A*δ*-related potentials, while 2 subjects had no clear vertex complex for the C fibers stimulation. Taking into consideration the averaged responses, the N2 and P2 latencies related to C-fibers had a mean delay of about 200 msec as compared to A*δ*-related waves, which is compatible with the C fibers-related delay [9] (Table 3). Amplitudes of C-related potentials were significantly reduced as compared to those of A*δ*-related responses (Table 3). The topographical representation of the C related N2 peak had a bilateral temporoparietal distribution, with a mild lateralization on the left side (Figure 1). The positive peak had a similar representation for the two modalities of stimulation (Figure 1). Considering the maximum positive peaks of single responses in the above-reported intervals, we confirmed a difference of latency between the 2 modalities of stimulation (Anova for repeated measures: F 644, DF 1, error DF 420 *p* < 0.0001). The difference in latency was independent from the series of stimulation (F 1,37 DF 20, and error DF 420 *p* 0.15).

**Figure 2:**
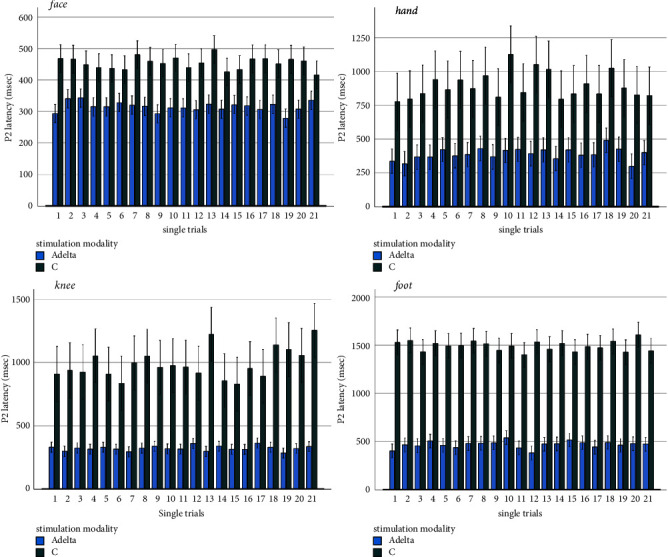
Mean values and standard errors of latencies of major amplitude positive peaks measurable in single trials in 20 healthy subjects with a-delta and C-modality of stimulation.

The confidence intervals of single responses among the 21 repetitions, varied from a minimum of 168 msec to a maximum of 322 msec for a-delta related responses, and from a minimum of 373 msec to a maximum of 539 msec for C-related P2 wave (Supplementary Table (available here)) (Figure 2).

### 3.2. Hand

**Figure 3:**
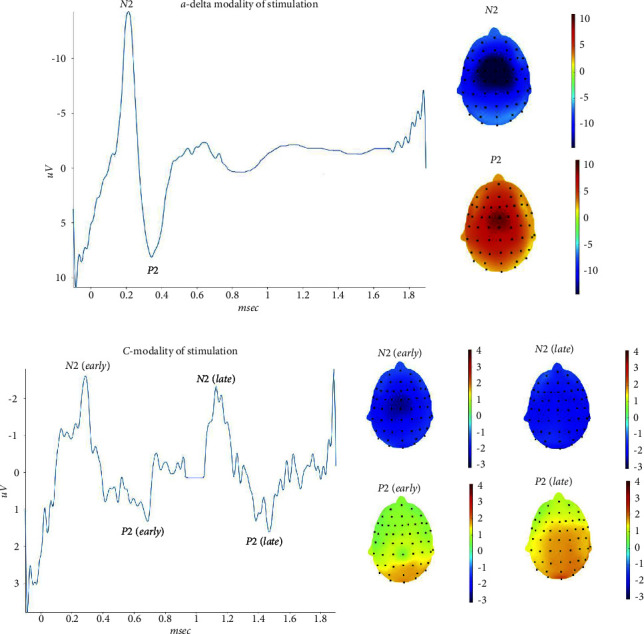
On the left side, the grand average of N2 P2 waves was obtained by right hand stimulation. On the right side, the corresponding topographical maps are represented. The grand average in the lower part of the figure, for the C-modality of stimulation, shows to distinct negative/positive (early and late) complexes. The topographical maps refers to the recorded N2-P2 waves. Upper: a-delta modality of stimulation. Lower: C-modality of stimulation.

The stimulation of the right hand elicited a clear averaged response in all the 20 subjects with the A*δ*-modality of stimulation. In 16 subjects, we found that the C stimulation modality elicited a positive response detectable from the background noise, preceded by a quite unclear negative wave. In 4 subjects we were not able to detect any response. In 12 subjects, we had a double complex, an earlier one in the first 650 msec, followed by a second late complex, in 4 subjects the average shows only a late complex. The grand-average calculated across all our subjects showed a first negative-positive complex in the first 650 msec, followed by a second complex in the 1–1.4 sec interval (Figure 3). The topographical distribution of the first negativity obtained with the C stimulation modality was similar to that obtained with A*δ*-stimulation. The early and late positive components obtained with the C fibers mode of stimulation were represented on the parietal sites (Figure 3).

We measured latency and amplitude in averaged responses, considering the negative-positive complex with the maximal amplitude, and found a significant prolongation of both N2 and P2 obtained with the C modality of stimulation, as compared with the A*δ* modality (Table 3).

In single trials, considering the maximum positive peaks detectable in the 2 seconds after stimulation, we confirmed a difference of latency between the 2 modalities of stimulation (Anova for repeated measures: F 785, DF 1, and error DF 418 *p* < 0.0001). The difference in latency was independent from the series of stimulation (F 1,31 DF 20, and error DF 418 *p* 0.16). The confidence intervals of single responses among the 21 repetitions varied from a minimum of 292 msec to a maximum of 401 msec for A*δ*-related responses, and from a minimum of 628 msec to a maximum of 1463 msec for C-related P2 wave (Supplementary Table (available here)) (Figure 3).

### 3.3. Knee

The stimulation of the right knee in the C-modality did not elicit a detectable response in 3 subjects, while in 10 of them, there was an early negative-positive in the first 500 msec, followed by a late complex in the 1–1.4 time interval.

**Figure 4:**
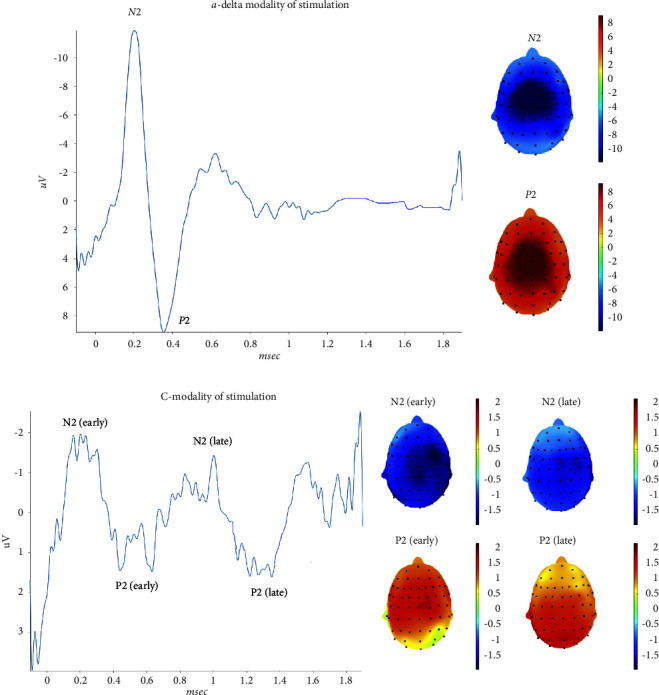
On the left side, the grand average of N2 P2 waves was obtained by right knee stimulation. On the right side, the corresponding topographical maps are represented. The grand average in the lower part of the figure, for the C-modality of stimulation, shows to distinct negative/positive (early and late) complexes. The topographical maps refers to the recorded N2-P2 waves. Upper: a-delta modality of stimulation. Lower: C-modality of stimulation.

As expected, latencies of late C-related N2 and P2 peaks were significantly prolonged as compared with those elicited with A*δ* modality, and amplitudes were also reduced (Table 3). The grand average of the vertex complex, showed an early negativity-positivity in the first 650 msec, followed by negative-positive waves in the 900–1250 msec interval (Figure 4). The early and late negative components evoked with the C-modality, had a wide scalp distribution, while the late positive wave was located posteriorly as compared to the P2 obtained with the A*δ* modality of stimulation (Figure 4).

Considering the single responses obtained with the C fibers modality of stimulation, as the maximum positive peak in the considered interval, we found that the positive peak latency varied in a range from 568 to 1335 msec (Supplementary Table, Figure 2). For the a-delta modality of stimulation, the latency range of P2 was 210–581 msec (Supplementary Table, Figure 2). The ANOVA for repeated measures, confirmed that latencies of the positive peak in single trials were significantly different for A*δ* and C fibers modality of stimulation (F 385.618 error DF 399 DF 1 *p* < 0.001). The series of stimulation was not relevant in the comparison between the two modalities of stimulation (F 0.47 DF 20 error DF 399 *p* 0.97).

### 3.4. Foot

The stimulation of the foot in the C fibers modality elicited a negative-positive complex in the first 650 msec in 10 subjects, followed by a larger late negative-positive response in the 1300–1900 msec interval. In 7 subject, we detected only a late response in the 1.100–1800 msec range. In 3 subject, we were not able to detect any response.

Latencies and amplitudes of maximal positive and negative peaks obtained with the 2 modalities of stimulation were significantly different (Table 3).

**Figure 5:**
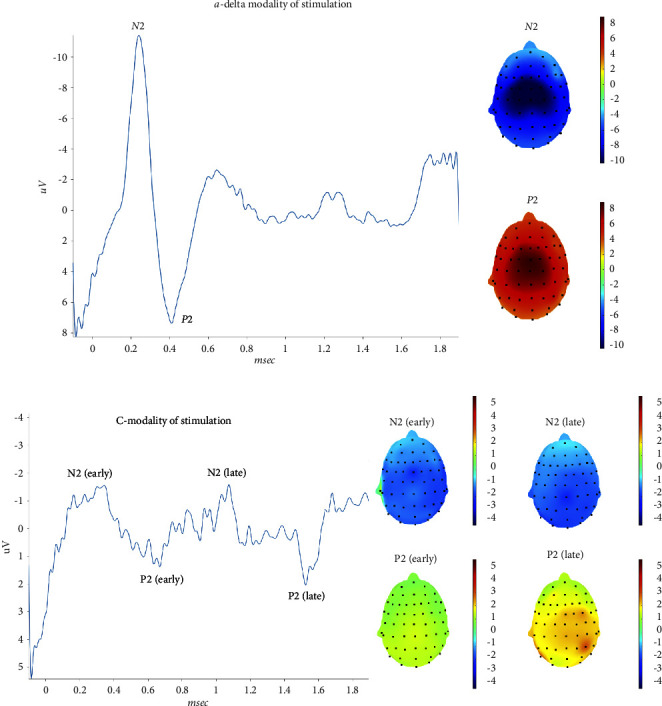
On the left side, the grand average of N2 P2 waves was obtained by right foot stimulation. On the right side, the corresponding topographical maps are represented. The grand average in the lower part of the figure, for the C-modality of stimulation, shows to distinct (early and late) negative/positive complexes. The topographical maps refer to the recorded N2-P2 waves. Upper: a-delta modality of stimulation. Lower: C-modality of stimulation.

The grand average obtained with the C fibers modality of stimulation showed a first negative-positive complex in the 350–650 msec interval, and a second one in the 1.1–1600 time range (Figure 5). The late negative peak was located posteriorly as compared to A*δ*-related N2. The late positive peak showed a shift toward the right parietal sites (Figure 5).

Single positive responses latency varied between 312–609 msec for the A*δ* modality of stimulation, and between 1330–1675 msec the for C-modality of stimulation.

The ANOVA for repeated measures confirmed that latencies of the positive peak in single trials were significantly different for a-delta and C fibers modality of stimulation (F 3963 error DF 336 DF 1 *p* < 0.001). The series of stimulation was not relevant in the comparison between the two modalities of stimulation (F 0.7 DF 20 error DF 336 *p* 0.78).

Values of latency of P2 components recorded in single trials (face, hand, knee, and foot) for the a-delta and C modalities of stimulation are reported in the Supplementary Table (available here).

Considering the latency of the averaged late positive peak obtained with the C fibers modality of stimulation at the foot and the knee, for a mean distance of 0.423 ± 0.05 meters and a mean latency difference of 0.401 ± 0.56 sec, we found a conduction velocity of 1.06 ± 1.9 m/sec.

Latencies and amplitudes of A*δ* and C fibers related responses did not show relevant differences between sexes.

## 4. Discussion

The present study aimed to define the reliability of ULEPs obtained with stimulation of both trigeminal and somatic sites. We used the ND : Yap laser, which has been largely applied in clinical practice for the evaluation of a-delta fibers function [14–16]. As for the C fibers modality of stimulation, we used that putatively selective for C thermoceptors, as described by Cruccu et al. [9]. This modality elicited a warm not burning sensation in all the healthy subjects. When we used the “standard” stimulation modality, suitable to activate the A*δ*-fibers, a clear painful pinprick was described by all our subjects. We paid particular attention to the individual sensations, and each subject was invited to alert the technician in case of a change of perception, especially when the stimulation parameters were adjusted to elicit a sensation of warmth. We observed a lower pinprick threshold in females, and a similar warmth threshold and A*δ* and C fibers related responses features in the two sexes. Gender related differences in pain processing is an important topic [17, 18], which deserve specific study in larger series. Whereas at the trigeminal level, we obtained a negative-positive complex in a time interval compatible with the C fibers activation, in the somatic districts the waves were hardly detectable, for a possible coactivation of the A*δ*-fibers. Nevertheless, a late positivity was measureable in most of the subjects for the upper and lower limb stimulation, allowing the computation of conduction velocity, which was in the range of C fibers.

In the following paragraphs, we describe and comment in detail main results.

### 4.1. Ultralate LEPs at Trigeminal Site

Healthy subjects reported a clear pinprick sensation stimulating the first trigeminal branch with the A*δ*-modality, and a clear warm nonpainful sensation with the C fibers modality of stimulation. We did not calculate conduction velocity in the trigeminal district, but ultralate latencies were just similar to those obtained by other groups, using the same modality of stimulation [9]. We observed that in 2 control subjects, whereas the A*δ*-related potentials were clearly evident, the C-related responses were absent, despite subjects felt warm sensation. This result could suggest that even at the trigeminal level, we can have a dispersion of signal conduction and that the use of these potentials for clinical purposes may be questionable [19]. In subjects with detectable responses after C-modality of stimulation, the latencies of the single trial responses varied in an interval compatible with C fibers conduction time. Moreover, a possible coactivation of afferents with different conduction velocity could not be excluded, as this could have generated a small not readable response in strict proximity to the thermal C-related potential. The topographic distribution of the negative wave seemed to slightly prevail on the left temporal regions as compared to the a-delta related potentials, the positive wave had a vertex distribution similar to the a-delta related P2. In previous studies, the dipolar source of trigeminal ULEPs was in the opercular regions for the early N1 and in the posterior cingulate for the negative-positive complex [9]. In the present study, focused on trigeminal and somatic U-LEPs, we did not take into consideration the early N1, for its low amplitude and hard detection from the noise, especially after limb stimulation. The ultralate negativity to trigeminal stimulation had a similar topographic distribution and possibly a similar origin as the A*δ*-related N1 [20]. Dipolar source analysis of ultralate responses could further explain the cortical origin of the negative component evoked with the C-modality of stimulation.

### 4.2. ULEPs at Hand, Knee, and Foot

After stimulation of the hand dorsum, knee, and foot, subjects felt a warm sensation for the C-fibers modality of stimulation and a painful pinprick in the case of A*δ* modality. Nevertheless, the grand average consisted of a low amplitude double negative-positive complex, the earlier preceding the later one by about 500–600 msec. The potentials obtained with the C-fibers modality of stimulation were absent in the 15–20% of subjects and, if evocable, were not clear and hardly readable as compared to the a-delta related potentials. Taking into consideration the maximum amplitude of the positive peaks, most patients exhibited the maximal peak in the time range compatible with the C-fibers conduction time. In fact, considering the distance between the stimulation site at the knee and foot, we calculated a conduction velocity of around 1 m/sec, which could satisfy the properties of the thermal C-fibers [21–23].

Our healthy controls had a high level of cooperation to the study, and they were instructed to signal if a feeling different from heat or warmth, depending on stimulation modality, could occur during LEP recording. The high level of attention to single stimuli is confirmed by the lack of habituation among single trials, a phenomenon described in previous studies for the A*δ* related LEPs and C-related U-LEPs [24–26] Single trials, in which we considered the maximum positive peak in the 2 seconds following the stimulus, confirmed that the stimulation in the C-fibers modality, evoked a maximum positivity in a time range compatible with the slow unmyelinated fibers conduction, despite the individual averages and the grand average showed earlier responses suggestive of coactivation of different types of fibers. We also found that latencies of late negative and positive waves evoked with the C-modality of stimulation varied within a wide range, another element in favor of a coactivation of afferents with different conduction velocity. In recent studies, the warm related potentials, evoked with a thermode stimulation from the trigeminal, hand, and foot sites were absent in more than 50% of 21 normal subjects at trigeminal level, and in most subjects after hand (13 subjects) and foot (18 subjects) stimulation [27]. These results could confirm that warm stimuli suitable for the activation of the innocuous warm receptors (TRPV3, TRPV4) produce an afferent volley subjected to a dispersion along the ascending pathways and possibly a coactivation of different fibers. All these elements may impair the readability of the signals. The early potential we observed in the majority of subjects, could be produced by the activation of high-threshold mechano-heat receptors (TRPV1) C fibers (CMH), and high-threshold mechano-heat receptors (TRPV1) Ad fibers (AMH-II), with a conduction velocity estimated respectively at 2.8 m/sec and 15 m/sec, as described by Magerl et al. [24]. Although no subject expressed a sensation of burning pain and/or noxious heat and pinprick, the activation threshold of such fibers could be very low, outside the edge of subjective perception.

The topographic distribution of the early and late positive peaks evoked from the hand and knee in the C stimulation modality showed a tendency through a more posterior distribution, as compared to the A*δ*-related potentials. After foot stimulation, we observed a similar posterior distribution for the later positivity. The negative component did not show clear and univocal localization on the scalp, confirming that for limbs stimulation, the late positive peak is more consistent than the negative one [28]. Garcia-Larrea et al. described a dipolar source of late positivity in the posterior part of the anterior cingulate, which was similar to the cortical generator of the P2 wave evoked with a-delta fibers stimulation [1], suggesting that the same cerebral areas are involved in both late and ultralate LEP generation.

## 5. Study Limits

This was a neurophysiological study aiming to reevaluate the features of ULEPs evoked with Nd : YAP laser in healthy subjects, in order to add elements about their reliability for possible clinical use. We did not compare LEPs with potentials evoked by other tools, such as thermodes, to activate innocuous warm related fibers. This could add important data to the hypothesis of a coactivation of different fibers, probably related to the specific modality of stimulation.

Although the late positivity in the averaged traces showed a different distribution for the A*δ* and C stimulation modality, we did not calculate the dipolar sources, thus possible different dipole location depending on stimulation modality remains speculative.

## 6. Conclusions

In this study, we confirmed that the stimulation with the Nd : YAP laser with the modality described by Truini et al. [2] is able to induce a warm not painful sensation in both the face and limbs. This subjective sensation did not correspond to the presence of clear cortical waves in all subjects even at the trigeminal level, where robust C-fibers related responses have been described [9, 10].

At the upper and lower limbs, the presence of an earlier negative-positive component preceding a later one suggested a coactivation of thermal receptors with a conduction velocity out of the classical C-fiber range. This phenomenon could be responsible for the hard detection of C-LEPs and could open a scenario about the low threshold of pain related heat receptors activation, outside the limit of subjective perception. At the trigeminal level, this coactivation of faster fibers could not be excluded, as the earlier potential could be in temporal proximity to the late one, and thus not detectable.

Moreover, single trials analysis allowed us to confirm that the maximal positive peak recordable after a Nd : YAP laser stimulation of low intensity, long duration, and large irradiated area, falls in the time interval of the low-threshold warm receptors.

We can just suppose the possible use of warm-related ULEPs with some cautions. Considering that in all of the healthy volunteers these potential were detectable in at least 2 of the 4 sites, their total absence could be a sign of systemic involvement of warm related C-fibers in patients with symptoms of small fibers neuropathy. Another possible application, could be the intrasubject comparison of ultralate warm potentials between the symptomatic and asymptomatic sites. In clinical setting, such ultralate warm potentials could be a complementary assessment to quantitative sensory testing (QST), skin biopsy, and a-delta related potentials.

Furthermore, studies will be addressed to the application of the such neurophysiological tool to patients with symptoms of systemic or localized small fibers impairment, to confirm or refuse the hypothesis of their possible clinical utility and reliability.

## Figures and Tables

**Table 1:** Pain thresholds for a-delta fibers modality of stimulation.

Patient number	Gender	Age (years)	Trig (J)	VAS	Hand (J)	VAS	Knee (J)	VAS	Foot (J)	VAS
1	F	64	2.50	90	2.25	28	2.75	90	2.75	57
2	F	22	1.75	90	2.00	84	2.25	90	2.25	75
3	M	53	2.75	75	2.75	62	2.50	63	3.00	75
4	F	23	2.25	66	3.25	47	2.50	49	2.75	63
5	F	51	2.50	90	2.50	75	3.75	75	4.00	75
6	F	22	2.00	65	2.00	60	2.50	70	2.25	66
7	F	24	2.00	80	2.75	86	2.25	73	2.25	77
8	F	41	2.50	50	3.75	75	3.50	80	3.50	73
9	M	49	2.25	83	3.25	87	3.00	80	2.50	90
10	F	50	2.50	79	3.50	90	4.00	65	3.50	86
11	M	27	2.25	70	2.75	76	2.75	70	3.75	85
12	F	31	2.50	60	2.75	69	3.00	48	3.50	70
13	M	47	3.50	68	3.50	60	2.75	53	3.00	84
14	F	23	2.00	85	2.50	90	2.50	80	2.25	95
15	F	22	1.75	90	2.50	75	3.00	80	3.00	75
16	M	43	3.00	70	3.75	64	3.50	60	3.75	70
17	F	26	2.25	60	2.75	79	2.75	27	3.25	80
18	M	41	2.75	60	3.25	70	3.00	55	4.00	55
19	M	29	3.00	68	4.00	30	3.50	51	5.00	70
20	F	31	2.75	63	3.00	75	4.00	90	3.75	70

(J: joules).

**Table 2:** Stimulation parameters of C-LEPs.

Patient number	Gender	Age (years)	Trig. (J)	Hand (J)	Knee (J)	Foot (J)	Presence U-LEP in stimulation sites
1	F	64	2.00	1.75	2.00	5.00	T-K-F
2	F	22	3.75	2.75	3.75	5.00	T-H-K-F
3	M	53	4.50	3.00	4.75	7.00	T-H-K-F
4	F	23	6.00	7.50	7.00	7.50	T-K-F
5	F	51	6.50	7.25	7.50	9.75	H-K-F
6	F	22	5.25	6.75	7.50	8.75	T-H-K-F
7	F	24	7.25	7.00	5.25	6.50	T-H-F
8	F	41	6.75	4.75	7.00	7.50	T-H-K-F
9	M	49	6.50	5.50	6.50	8.25	T-K
10	F	50	6.50	7.25	6.00	7.50	T-H-K-F
11	M	27	5.50	6.25	7.00	7.75	T-H-K-F
12	F	31	7.50	8.25	9.75	10.00	H-K-F
13	M	47	7.25	7.25	8.25	8.00	T-H-K
14	F	23	6.00	6.25	6.25	6.25	T-H-F
15	F	22	6.75	8.00	8.25	9.25	T-H-K-F
16	M	43	8.25	8.25	8.75	9.25	T-H-K-F
17	F	26	5.25	7.25	7.00	10.25	T-K-F
18	M	41	5.50	6.75	7.50	7.00	T-H-K-F
19	M	29	7.25	8.75	8.50	10.50	T-H-K
20	F	31	5.25	5.25	5.25	6.50	T-H-F

(J: joules, T: trigeminal site, K: knee, H: hand, and F: foot).

**Table 3:** Values of latencies and amplitudes of N2P2 components for the a-delta and C modalities of stimulation in 20 healthy subjects. Results of statistical analysis are reported.

		Stimulation type	*N*	Mean	SD	Error ds	Student's *t* test	*p*	Leven test (*F*)	*p*
Face	N2 latency (msec)	Ad	20	161.00	19.167	4.286	−6.95	<0.001	15.77	<0.001
		C	18	283.52	76.283	17.980				
	P2 latency (msec)	Ad	20	330.08	51.120	11.431	−6.76	<0.001	6.61	0.014
		C	18	493.51	93.751	22.097				
	N2 amplitude (uV)	Ad	20	−17.14	9.81	2.19	4.96	<0.001	9.21	0.004
		C	18	−5.42	2.09	0.49				
	P2 amplitude (uV)	Ad	20	9.31	5.15	1.15	6.61	<0.001	6.61	0.014
		C	18	3.78	2.76	0.65				
Hand	N2 latency (msec)	Ad	20	228.33	57.90	13.28	3.23	0.001	21.72	<0.001
		C	16	455.42	276.14	69.03				
	P2 latency (msec)	Ad	20	345.31	34.05	7.81	−8.37	<0.001	35.74	<0.001
		C	16	1062.84	355.73	88.93				
	N2 amplitude (uV)	Ad	20	−17.04	11.13	2.55	−3.54	<0.001	6.59	0.02
		C	16	−7.18	4.37	1.09				
	P2 amplitude (uV)	Ad	20	10.95	5.71	1.31	2.04	0.025	0.29	0.58
		C	16	7.26	4.89	1.22				
Knee	N2 latency (msec)	Ad	20	206.64	22.76	5.09	−7	<0.001	30.83	<0.001
		C	17	1026.84	431.91	104.75				
	P2 latency (msec)	Ad	20	374.22	36.28	8.11	11.89	<0.001	29.55	<0.001
		C	17	1319.12	325.91	79.05				
	N2 amplitude (uV)	Ad	20	−14.83	9.36	2.09	-3.11	0.002	8.37	0.007
		C	17	−7.33	4.92	1.19				
	P2 amplitude (uV)	Ad	20	8.10	5.42	1.21	1.75	0.045	7.97	0.008
		C	17	5.76	2.31	0.56				
Foot	N2 latency (msec)	Ad	20	243.36	27.47	6.14	−12.34	<0.001	21.85	<0.001
		C	17	1048.44	267.77	64.94				
	P2 latency (msec)	Ad	20	400.20	82.64	18.48	−13.16	<0.001	18.37	<0.001
		C	17	1348.52	287.01	69.61				
	N2 amplitude (uV)	Ad	20	−14.06	9.00	2.01	−2.88	0.006	2.05	0.61
		C	17	−6.91	5.22	1.27				
	P2 amplitude (uV)	Ad	20	9.75	4.68	1.05	2.76	0.006	0.12	0.72
		C	17	5.95	3.85	0.93				

## Data Availability

The data are available upon request.

## Abbreviations

ISI: Inter-stimulus interval
LEPs: Laser evoked potentials
Nd : YAP Laser: Neodymium : yttrium-aluminium-perovskite laser
QST: Quantitative sensory testing
SII: Secondary somatosensory cortex
ULEPs: Ultralate LEPs
VAS: Visual-analogue scale.

## Supplementary Materials

Supplementary Materials Supplementary Table. Values of latency of P2 components recorded in single trials (face, hand, knee, and foot) for the a-delta and C-modalities of stimulation. Deviation standard (DS) and confidence interval (CI) are reported.
