# Supplementary material for: Assessment of C Fibers Evoked Potentials in Healthy Subjects by Nd : YAP Laser
Source: Pain Res Manag. 2022 Dec 26;2022:7737251. doi: 10.1155/2022/7737251 (PMC9807303; doi:10.1155/2022/7737251)
Supplement: Supplementary Materials — Supplementary Table. Values of latency of P2 components recorded in single trials (face, hand, knee, and foot) for the a-delta and C-modalities of stimulation. Deviation standard (DS) and confidence interval (CI) are reported. [file 7737251.f1.docx]

Suppl. Tab. - Values of latency of P2 components recorded in single trials (face, hand, knee and foot) for the a-delta and C modalities of stimulation. Deviation Standard (DS) and Confidence Interval (CI) are reported.

| single trial  (face) | | Mean | Error DS. | CI 95% | | single trial  (hand) | | Mean | Error DS. | CI 95% | | single trial  (Knee) | | Mean | Error DS. | CI 95% | | single trial  (foot) | | Mean | Error DS. | CI 95% | |
| --- | --- | --- | --- | --- | --- | --- | --- | --- | --- | --- | --- | --- | --- | --- | --- | --- | --- | --- | --- | --- | --- | --- | --- |
|  |  |  |  |  |  |  |  |  |  |  |  |  |  |  |  |  |  |  |  |  |  |  |  |
| 1 | Ad | 0.214 | 0.018 | 0.179 | 0.250 | 1 | Ad | 0.331 | 0.020 | 0.292 | 0.371 | 1 | Ad | 337.03 | 45.27 | 248.03 | 426.03 | 1 | Ad | 402.99 | 35.23 | 333.68 | 472.30 |
|  | C | 0.440 | 0.024 | 0.393 | 0.487 |  | C | 0.908 | 0.109 | 0.694 | 1.123 |  | C | 776.56 | 105.72 | 568.71 | 984.40 |  | C | 1528.45 | 64.14 | 1402.28 | 1654.61 |
| 2 | Ad | 0.214 | 0.018 | 0.178 | 0.250 | 2 | Ad | 0.298 | 0.020 | 0.259 | 0.338 | 2 | Ad | 317.97 | 45.27 | 228.97 | 406.97 | 2 | Ad | 465.26 | 35.23 | 395.95 | 534.57 |
|  | C | 0.416 | 0.024 | 0.369 | 0.463 |  | C | 0.937 | 0.109 | 0.722 | 1.151 |  | C | 795.90 | 105.72 | 588.05 | 1003.75 |  | C | 1549.59 | 64.14 | 1423.43 | 1675.75 |
| 3 | Ad | 0.211 | 0.018 | 0.176 | 0.247 | 3 | Ad | 0.325 | 0.020 | 0.287 | 0.364 | 3 | Ad | 367.56 | 45.27 | 278.56 | 456.56 | 3 | Ad | 455.61 | 35.23 | 386.30 | 524.92 |
|  | C | 0.409 | 0.024 | 0.362 | 0.457 |  | C | 0.925 | 0.106 | 0.715 | 1.134 |  | C | 836.71 | 105.72 | 628.87 | 1044.56 |  | C | 1429.64 | 64.14 | 1303.47 | 1555.80 |
| 4 | Ad | 0.218 | 0.018 | 0.182 | 0.253 | 4 | Ad | 0.315 | 0.020 | 0.276 | 0.354 | 4 | Ad | 366.92 | 45.27 | 277.92 | 455.92 | 4 | Ad | 505.01 | 35.23 | 435.70 | 574.32 |
|  | C | 0.436 | 0.024 | 0.389 | 0.483 |  | C | 1.053 | 0.106 | 0.844 | 1.263 |  | C | 941.01 | 105.72 | 733.17 | 1148.86 |  | C | 1520.62 | 64.14 | 1394.46 | 1646.79 |
| 5 | Ad | 0.208 | 0.018 | 0.173 | 0.244 | 5 | Ad | 0.331 | 0.020 | 0.292 | 0.370 | 5 | Ad | 422.12 | 45.27 | 333.12 | 511.12 | 5 | Ad | 459.51 | 35.23 | 390.20 | 528.82 |
|  | C | 0.400 | 0.024 | 0.353 | 0.447 |  | C | 0.909 | 0.106 | 0.699 | 1.118 |  | C | 867.58 | 105.72 | 659.73 | 1075.43 |  | C | 1493.07 | 64.14 | 1366.91 | 1619.23 |
| 6 | Ad | 0.201 | 0.018 | 0.166 | 0.237 | 6 | Ad | 0.315 | 0.020 | 0.276 | 0.354 | 6 | Ad | 376.83 | 45.27 | 287.84 | 465.83 | 6 | Ad | 437.00 | 35.23 | 367.69 | 506.30 |
|  | C | 0.475 | 0.024 | 0.428 | 0.522 |  | C | 0.837 | 0.106 | 0.628 | 1.047 |  | C | 938.48 | 105.72 | 730.63 | 1146.32 |  | C | 1496.51 | 64.14 | 1370.35 | 1622.67 |
| 7 | Ad | 0.215 | 0.018 | 0.180 | 0.251 | 7 | Ad | 0.294 | 0.020 | 0.255 | 0.333 | 7 | Ad | 385.23 | 45.27 | 296.23 | 474.23 | 7 | Ad | 479.50 | 35.23 | 410.20 | 548.81 |
|  | C | 0.401 | 0.024 | 0.354 | 0.448 |  | C | 0.998 | 0.106 | 0.789 | 1.207 |  | C | 873.64 | 105.72 | 665.79 | 1081.49 |  | C | 1544.53 | 64.14 | 1418.37 | 1670.69 |
| 8 | Ad | 0.223 | 0.018 | 0.188 | 0.259 | 8 | Ad | 0.324 | 0.020 | 0.285 | 0.363 | 8 | Ad | 431.50 | 45.27 | 342.50 | 520.50 | 8 | Ad | 481.34 | 35.23 | 412.03 | 550.65 |
|  | C | 0.491 | 0.024 | 0.444 | 0.539 |  | C | 1.052 | 0.106 | 0.842 | 1.261 |  | C | 969.92 | 105.72 | 762.08 | 1177.77 |  | C | 1515.11 | 64.14 | 1388.95 | 1641.27 |
| 9 | Ad | 0.287 | 0.018 | 0.251 | 0.322 | 9 | Ad | 0.338 | 0.020 | 0.299 | 0.376 | 9 | Ad | 368.99 | 45.27 | 279.99 | 457.99 | 9 | Ad | 487.09 | 35.23 | 417.78 | 556.39 |
|  | C | 0.438 | 0.024 | 0.391 | 0.485 |  | C | 0.961 | 0.106 | 0.751 | 1.170 |  | C | 811.52 | 105.72 | 603.68 | 1019.37 |  | C | 1445.72 | 64.14 | 1319.56 | 1571.89 |
| 10 | Ad | 0.206 | 0.018 | 0.171 | 0.242 | 10 | Ad | 0.317 | 0.020 | 0.279 | 0.356 | 10 | Ad | 417.38 | 45.27 | 328.38 | 506.37 | 10 | Ad | 539.93 | 35.23 | 470.63 | 609.24 |
|  | C | 0.448 | 0.024 | 0.401 | 0.495 |  | C | 0.975 | 0.106 | 0.766 | 1.184 |  | C | 1127.15 | 105.72 | 919.30 | 1335.00 |  | C | 1493.06 | 64.14 | 1366.90 | 1619.22 |
| 11 | Ad | 0.259 | 0.018 | 0.224 | 0.295 | 11 | Ad | 0.315 | 0.020 | 0.277 | 0.354 | 11 | Ad | 423.44 | 45.27 | 334.44 | 512.44 | 11 | Ad | 433.78 | 35.23 | 364.47 | 503.09 |
|  | C | 0.461 | 0.024 | 0.414 | 0.508 |  | C | 0.965 | 0.106 | 0.755 | 1.174 |  | C | 845.50 | 105.72 | 637.66 | 1053.35 |  | C | 1398.38 | 64.14 | 1272.22 | 1524.55 |
| 12 | Ad | 0.250 | 0.018 | 0.215 | 0.286 | 12 | Ad | 0.359 | 0.020 | 0.320 | 0.398 | 12 | Ad | 392.74 | 45.27 | 303.74 | 481.73 | 12 | Ad | 382.08 | 35.23 | 312.77 | 451.39 |
|  | C | 0.420 | 0.024 | 0.373 | 0.467 |  | C | 0.916 | 0.106 | 0.706 | 1.125 |  | C | 1051.95 | 105.72 | 844.10 | 1259.80 |  | C | 1531.89 | 64.14 | 1405.73 | 1658.05 |
| 13 | Ad | 0.213 | 0.018 | 0.178 | 0.249 | 13 | Ad | 0.298 | 0.020 | 0.259 | 0.336 | 13 | Ad | 420.66 | 45.27 | 331.66 | 509.66 | 13 | Ad | 473.76 | 35.23 | 404.45 | 543.07 |
|  | C | 0.432 | 0.024 | 0.385 | 0.479 |  | C | 1.224 | 0.106 | 1.015 | 1.434 |  | C | 1016.02 | 105.72 | 808.17 | 1223.87 |  | C | 1459.06 | 64.14 | 1332.90 | 1585.22 |
| 14 | Ad | 0.216 | 0.018 | 0.181 | 0.252 | 14 | Ad | 0.338 | 0.020 | 0.300 | 0.377 | 14 | Ad | 356.14 | 45.27 | 267.15 | 445.14 | 14 | Ad | 475.37 | 35.23 | 406.06 | 544.68 |
|  | C | 0.490 | 0.024 | 0.443 | 0.537 |  | C | 0.856 | 0.106 | 0.647 | 1.066 |  | C | 795.51 | 105.72 | 587.66 | 1003.35 |  | C | 1520.41 | 64.14 | 1394.24 | 1646.57 |
| 15 | Ad | 0.212 | 0.018 | 0.176 | 0.247 | 15 | Ad | 0.314 | 0.020 | 0.275 | 0.353 | 15 | Ad | 420.53 | 45.27 | 331.54 | 509.53 | 15 | Ad | 516.73 | 35.23 | 447.42 | 586.04 |
|  | C | 0.433 | 0.024 | 0.386 | 0.481 |  | C | 0.828 | 0.106 | 0.619 | 1.037 |  | C | 834.96 | 105.72 | 627.12 | 1042.81 |  | C | 1428.95 | 64.14 | 1302.78 | 1555.11 |
| 16 | Ad | 0.209 | 0.018 | 0.174 | 0.245 | 16 | Ad | 0.313 | 0.020 | 0.274 | 0.352 | 16 | Ad | 381.25 | 45.27 | 292.25 | 470.25 | 16 | Ad | 488.46 | 35.23 | 419.16 | 557.77 |
|  | C | 0.439 | 0.024 | 0.392 | 0.486 |  | C | 0.953 | 0.106 | 0.743 | 1.162 |  | C | 910.16 | 105.72 | 702.31 | 1118.01 |  | C | 1484.78 | 64.14 | 1358.62 | 1610.95 |
| 17 | Ad | 0.214 | 0.018 | 0.178 | 0.250 | 17 | Ad | 0.362 | 0.020 | 0.324 | 0.401 | 17 | Ad | 384.08 | 45.27 | 295.08 | 473.08 | 17 | Ad | 442.05 | 35.23 | 372.74 | 511.36 |
|  | C | 0.391 | 0.024 | 0.344 | 0.439 |  | C | 0.891 | 0.106 | 0.681 | 1.100 |  | C | 834.95 | 105.72 | 627.11 | 1042.80 |  | C | 1472.83 | 64.14 | 1346.67 | 1598.99 |
| 18 | Ad | 0.223 | 0.018 | 0.187 | 0.258 | 18 | Ad | 0.331 | 0.020 | 0.292 | 0.369 | 18 | Ad | 492.19 | 45.27 | 403.19 | 581.18 | 18 | Ad | 490.07 | 35.23 | 420.77 | 559.38 |
|  | C | 0.437 | 0.024 | 0.390 | 0.484 |  | C | 1.138 | 0.106 | 0.928 | 1.347 |  | C | 1026.77 | 105.72 | 818.92 | 1234.61 |  | C | 1539.25 | 64.14 | 1413.09 | 1665.42 |
| 19 | Ad | 0.235 | 0.018 | 0.200 | 0.271 | 19 | Ad | 0.285 | 0.020 | 0.247 | 0.324 | 19 | Ad | 425.51 | 45.27 | 336.51 | 514.51 | 19 | Ad | 462.73 | 35.23 | 393.42 | 532.04 |
|  | C | 0.436 | 0.024 | 0.389 | 0.483 |  | C | 1.102 | 0.106 | 0.893 | 1.312 |  | C | 877.54 | 105.72 | 669.69 | 1085.38 |  | C | 1427.11 | 64.14 | 1300.95 | 1553.27 |
| 20 | Ad | 0.226 | 0.018 | 0.190 | 0.261 | 20 | Ad | 0.319 | 0.020 | 0.281 | 0.358 | 20 | Ad | 299.39 | 45.27 | 210.39 | 388.39 | 20 | Ad | 476.98 | 35.23 | 407.67 | 546.28 |
|  | C | 0.440 | 0.024 | 0.393 | 0.487 |  | C | 1.057 | 0.106 | 0.848 | 1.267 |  | C | 828.52 | 105.72 | 620.67 | 1036.36 |  | C | 1608.64 | 64.14 | 1482.48 | 1734.80 |
| 21 | Ad | 0.204 | 0.018 | 0.168 | 0.239 | 21 | Ad | 0.336 | 0.020 | 0.297 | 0.374 | 21 | Ad | 400.54 | 45.27 | 311.54 | 489.54 | 21 | Ad | 472.84 | 35.23 | 403.53 | 542.15 |
|  | C | 0.466 | 0.024 | 0.419 | 0.513 |  | C | 1.254 | 0.106 | 1.045 | 1.463 |  | C | 822.26 | 105.72 | 614.42 | 1030.11 |  | C | 1441.13 | 64.14 | 1314.97 | 1567.29 |
